# Supplementary material for: Clinicopathological Features and Survival of Patients with Hepatocellular Carcinoma in Ethiopia: A Multicenter Study
Source: Cancers (Basel). 2022 Dec 28;15(1):193. doi: 10.3390/cancers15010193 (PMC9818669; doi:10.3390/cancers15010193)
Supplement: Supplementary file 1 [file cancers-15-00193-s001.zip › cancers-2067781-supplementary.pdf]

cancers

MDPI

Supplementary materials

Table S1. Barcelona Clinic Liver Cancer (BCLC) staging system adopted from the NCCN guideline.

NCCN National Comprehensive Cancer Network®

NCCN Guidelines Version 3.2022
Hepatobiliary Cancers

Barcelona Clinic Liver Cancer (BCLC) Staging System (2022)[1]

***Table 1.* Definitions for Prognostic Groups**

Stage	Definition
Very early stage (0)	• Single ≤2 cm<br>• Preserved liver function,[a] PS 0
Early stage (A)	• Single, or ≤3 nodules each ≤3 cm<br>• Preserved liver function,[a] PS 0
Intermediate stage (B)	• Multinodular<br>• Preserved liver function,[a] PS 0
Advanced stage (C)	• Portal invasion and/or extrahepatic spread<br>• Preserved liver function, PS 1-2
Terminal stage (D)	• Any tumor burden<br>• End stage liver function, PS 3-4

[a] Except for those with tumor burden acceptable for transplant.

Referenced with permission from the NCCN Clinical Practice Guidelines in Oncology (NCCN Guidelines®) for Hepatobiliary Cancers Version.3.2022. © National Comprehensive Cancer Network, Inc. 2022. All rights reserved. Accessed [December 09, 2022]

Table S2. The differences in overall survival (OS) of HCC patients among the study centers.

Study centers	HCC patients in the study (n)	Cirrhotic patients (N (%))	Median OS (days)	95% CI (Range, days)
TASH	200	124 (62)	119	94 – 144
SPHMMC	89	45 (50)	185	118 – 252
HUCSH	57	32 (56)	205	151 – 259
JUMC	23	12 (52)	77	64 – 90

TASH: Tikur Anbessa Specialized Hospital, SPHMMC: St. Paul's Hospital Millennium Medical College, HUCSH: Hawassa University Comprehensive Specialized Hospital, JUMC: Jimma University Medical Center. HCC: hepatocellular carcinoma. OS: overall survival

- We have observed differences in survival of HCC patients among the study centers. The median OS were significantly shorter for patients recruited from TASH and JUMC as compared to HUCSH and SPHMMC. The differences might be due to variations in quality of care/medical services as well as patients' condition. As shown in Table S2, the higher number of cirrhotic cases in TASH (> 60%) may have contributed to low survival although this center is known to provide a relatively good quality of oncology service. There may also be additional factors that needs further investigation.
- Nevertheless, it is difficult to compare one center over the other, certainly when considering the large differences in number of patients from various centers. Please note that about 80% of study participants were recruited from the two tertiary referral oncology care centers, TASH and SPHMMC.
